# β-Klotho Promotes the Development of Intrauterine Adhesions via the PI3K/AKT Signaling Pathway

**DOI:** 10.3390/ijms231911294

**Published:** 2022-09-25

**Authors:** Zizhen Guo, Yuqing Wang, Xiaoyang Wen, Xinxin Xu, Lei Yan

**Affiliations:** 1 Center for Reproductive Medicine, Shandong University, Jinan 250000, China; 2 Key Laboratory of Reproductive Endocrinology of Ministry of Education, Shandong University, Jinan 250000, China; 3 Shandong Key Laboratory of Reproductive Medicine, Jinan 250000, China; 4 The First Clinical College, Shandong University of Traditional Chinese Medicine, Jinan 250000, China; 5 Reproductive and Genetic Center of Integrative Medicine, Affiliated Hospital of Shandong University of Traditional Chinese Medicine, Jinan 250000, China

**Keywords:** intrauterine adhesion, β-Klotho, PI3K/AKT, fibrosis

## Abstract

Intrauterine adhesion (IUA) refers to injury to the basal layer of the endometrium, which can be caused by various factors. It is often accompanied by clinical symptoms such as abnormal menstruation, infertility, recurrent abortion, and periodic abdominal pain. In recent years, a number of studies have reported the effects of β-Klotho (KLB) on the occurrence and development of human tumors and fibrotic diseases, but its relationship with endometrial fibroblasts and endometrial fibrosis has not been elucidated. In this study, we compared the expression of KLB in endometrial stromal cells (ESCs) from patients with IUA and normal controls. We constructed animal and cell models of IUA and conducted expression verification and functional experiments on KLB. We found that the expression of KLB was significantly increased in the ESCs of IUA patients and rat models compared with the controls. The overexpression of KLB could promote the proliferation and fibrosis of ESCs. In addition, the overexpression of KLB activated the PI3K/AKT signaling pathway in ESCs. Our study shows that KLB protein is highly expressed in the ESCs of patients with IUA and can enhance stromal cell proliferation and cell fibrosis by activating the PI3K/AKT pathway, thus promoting the development of IUA.

## 1. Introduction

Intrauterine adhesion (IUA) refers to injury to the basal layer of the endometrium, which can be caused by various factors [1]. In the process of wound healing, scars or adhesive bands are formed on the surface of the endometrium, resulting in the partial or complete occlusion of the uterine cavity. The pathogenesis of IUA is still not very clear. Scholars have put forward the following five theories: trauma [2], infection [3], fibroblast proliferation [4], stem cell damage [5], and neural reflex [6]. Among these, trauma has always been recognized as the main factor causing uterine adhesion, and endometrial fibrosis is the main pathological feature and final result of uterine adhesion.

TGF-β1 has long been regarded as the central mediator of fibrosis. This cytokine induces fibroblasts to activate and secrete extracellular matrix (ECM). Several studies have reported an increase in the area of endometrial fibrosis in IUA patients and animal models, along with an abnormal up-regulation of the expression of TGF-β1 as a marker of fibrosis [7,8]. In addition to the classical signal pathways mediated by mothers against decapentaplegic (Smad), TGF-β1 can activate non-classical signaling pathways. For example, the phosphatidylinositol 3-kinase/protein kinase B (PI3K/AKT) signaling pathway has been reported to be activated by TGF-β1, independently of Smad2 and Smad3, and to modulate fibroblast responses [9]. In addition, the PI3K/Akt signaling pathway is involved in many important cellular processes, including cell survival, apoptosis, proliferation, and differentiation [10]. 

Klotho was initially considered to be a powerful anti-aging gene. In animal studies, it has been observed that Klotho deficiency can lead to a variety of aging phenotypes, including severe growth retardation, premature organ degeneration, arteriosclerosis, and the subsequent shortening of life expectancy [11]. The Klotho family is a newly defined protein family composed of α-Klotho (KLA), β-Klotho (KLB), and γ-Klotho. Klotho proteins are type I transmembrane proteins that are very important for mediating transmembrane signal transduction. Zhu et al. showed that KLA may inhibit the development of renal cell carcinoma by inhibiting PI3K/AKT/GSK3β/Snail signaling [12]. Meanwhile, FGF23 signaling requires KLA as a coreceptor in organs, where it regulates phosphate homeostasis [13]. Jarrod W Barnes et al. reported that the down-regulation of KLA may lead to fibrosis and inflammation in idiopathic pulmonary fibrosis, while FGF23 may act as a compensatory antifibrotic and anti-inflammatory mediator by inhibiting TGF-β signaling [14]. KLB has 41% homology with KLA in its amino acid sequence [15].

In recent years, several studies have been conducted on the effects of KLB on the occurrence and development of various human tumors and fibrotic diseases. It has been confirmed that KLB affects the occurrence and development of liver fibrosis, liver cancer, lung cancer, thyroid cancer, etc. Specifically, KLB has been identified as a tumor suppressor in prostate cancer [16], thyroid cancer [17], and lung cancer [18]. On the contrary, KLB promotes the growth of bladder cancer [19] and has carcinogenic activity in hepatocellular carcinoma [20] and hepatoblastoma [21]. Paradoxically, KLB has also been shown to inhibit the growth of hepatocellular carcinoma associated with fibroblast growth factor receptor 4 [22]. In addition, the down-regulation of KLB was found in the liver tissues of children with nonalcoholic fatty liver disease [23] and adults with liver fibrosis [24]. Therefore, KLB plays different roles in different tissues and cells, but there are no reports about its relationship with endometrial fibroblasts and endometrial fibrosis.

In this study, we compared endometrial fibroblasts from patients with IUA and normal controls and found an increased expression of KLB in fibroblasts from patients with IUA. We also preliminarily explored the potential association between the KLB/PI3k/AKT pathway and endometrial fibrosis.

## 2. Results

### 2.1. KLB Is Up-Regulated in ESCs of IUA Patients

**Figure 1:**
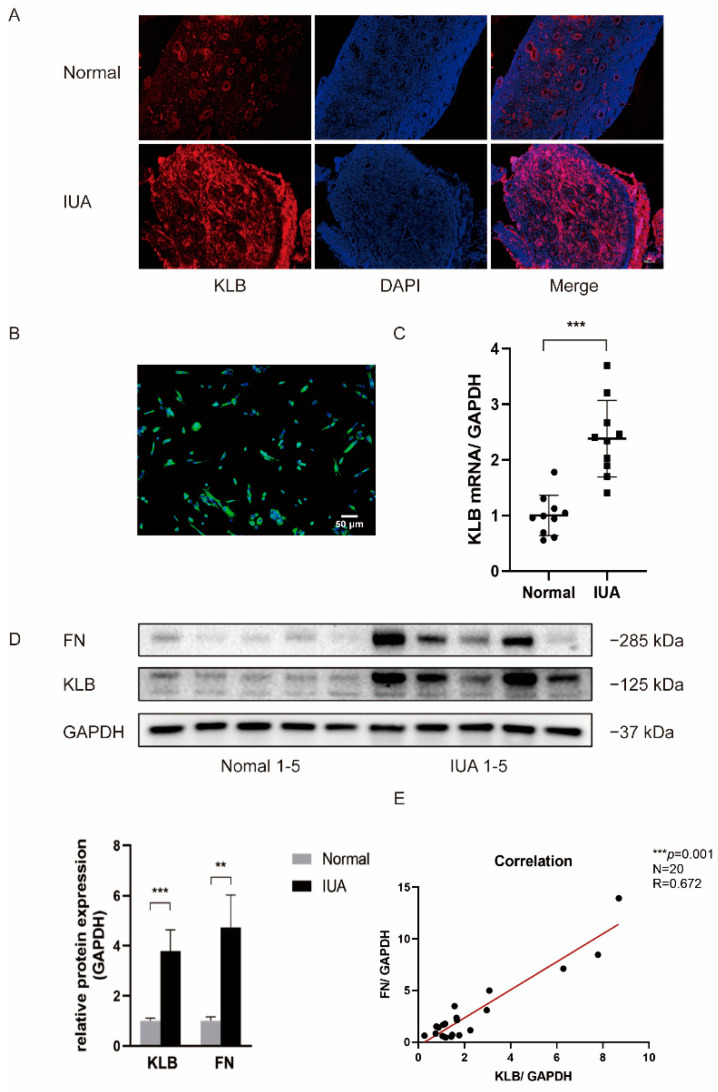
KLB expression was elevated in ESCs of patients with IUA. (**A**) KLB was expressed in both stromal and epithelial cells. Compared with the normal control group, the expression of KLB in ESCs of patients with IUA was increased. Scale bar = 100 μm. (**B**) The primary ESCs were extracted and identified with Vimentin. Immunofluorescence showed that the positive rate of Vimentin was more than 96%. Scale bar = 50 μm. (**C**) Through RT-qPCR analysis, it was found that the expression of KLB mRNA in the primary ESCs of the IUA group was higher than in those of the normal control group. (**D**) The expression of KLB and the fibrosis marker Fibronectin (FN) in primary ESCs of the IUA group and normal control group was detected and analyzed using Western blotting. It was found that the expression of KLB and FN increased in the primary ESCs of IUA patients. (**E**) Spearman rank correlation analysis showed that the expressions of KLB and FN proteins in ESCs were positively correlated. ** *p* < 0.01; *** *p* ≤ 0.001.

We used immunofluorescence, Western blotting, and RT-qPCR to determine the difference in KLB expression in the ESCs of patients with and without IUA. Firstly, the location and expression levels of KLB protein in the endometrium of IUA patients and non-IUA patients were detected using an immunofluorescence technique. We found that KLB protein expression increased in the ESCs of IUA patients (Figure 1A). Next, we extracted primary cells from fresh endometrial tissue and isolated primary stromal cells. The specific marker Vimentin of ESCs was identified by immunofluorescence staining, and the positive rate of Vimentin was over 96% (Figure 1B), indicating that the isolated cells were stromal cells. Total protein and total RNA were extracted from primary ESCs, and KLB expression was detected in 10 pairs of ESCs from IUA and normal non-IUA patients. The results showed that KLB mRNA (Figure 1C) and KLB protein (Figure 1D) expression was significantly increased in IUA patients. Meanwhile, we detected the expression of fibronectin in the primary ESCs of IUA patients and normal controls, and the results showed that the expression of fibronectin in IUA patients was significantly increased (Figure 1E). It was observed that the expression of KLB and fibronectin increased in the primary ESCs of patients. Spearman’s rank correlation analysis showed that there was a positive correlation between the expression levels of KLB and fibronectin in primary ESCs.

### 2.2. Construction of an IUA Animal Model and Verification of KLB Expression

**Figure 2:**
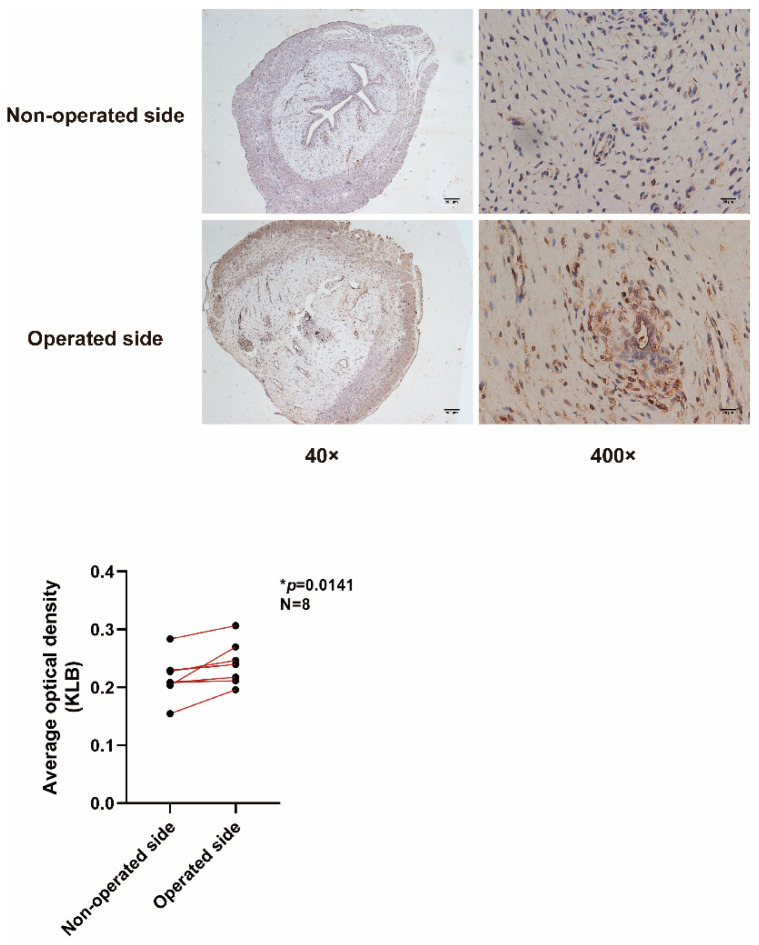
IHC to detect KLB in rats. The lumen surface of the uterus on the operative side (IUA side) was discontinuous, the uterine lumen was narrowed, and the glands of the damaged uterus were decreased. KLB was expressed in both epithelial and stromal cells of the rats, and KLB staining on the operative side was stronger than on the non-operative side. Statistical analysis of eight pairs of rat endometrial sections (400×) with ESCs showed significant differences (*p* = 0.0141).

Endometrial tissue samples from IUA rats were stained by IHC, which showed that the lumen surface of the uterus on the operative side (IUA side) was discontinuous, the uterine lumen narrowed, and the glands of the damaged uterus decreased (Figure 2). KLB was expressed in both epithelial and stromal cells of the rats, and KLB staining on the operative side was stronger than on the non-operative side. Statistical analysis of eight pairs of rat endometrial sections (400×) with ESCs showed a significant difference (*p* = 0.0141).

### 2.3. Construction of an IUA Cell Model and Verification of KLB Expression

**Figure 3:**
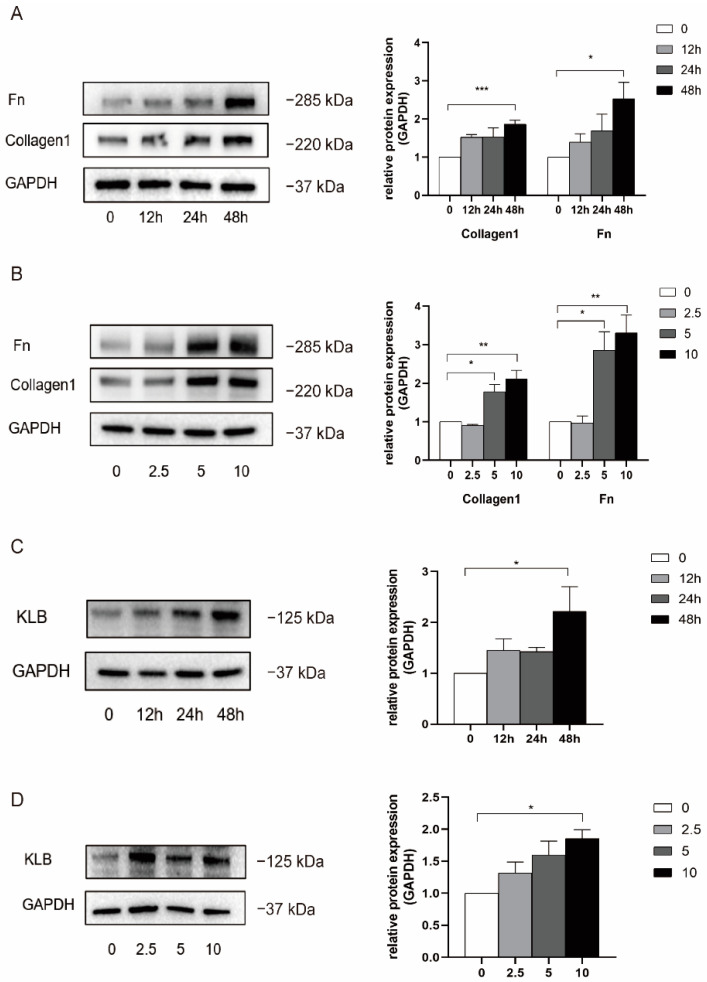
TGF-β1 was used to stimulate HESCs to construct an IUA cell model and detect the expression of KLB. (**A**) Western blot was used to detect the expression of Collagen I and FN in HESCs stimulated with TGF-β1 at the concentration of 10 ng/mL for 0, 12, 24, and 48 h. It was found that the expression of Collagen I and FN increased in a statistically significant manner when treated with 10 ng/mL for 48 h. (**B**) The expression of Collagen I and FN in HESCs stimulated with TGFβ1 at the concentrations of 0, 2.5, 5, and 10 ng/mL for 48 h was detected by Western Blot. It was found that the expression of Collagen I and FN was significantly increased when the cells were treated with 5 ng/mL and 10 ng/mL. (**C**) The expression of KLB in HESCs stimulated with TGF-β1 at the concentration of 10 ng/mL was detected by Western Blot, and it was found that the expression of KLB increased after 48 h of TGF-β1 stimulation. (**D**) The expression of KLB in HESCs stimulated with TGF-β1 at the concentrations of 0, 2.5, 5, and 10 ng/mL for 48 h was detected by Western Blot. It was found that the expression of KLB in HESCs increased when the cells were stimulated with TGF-β1 at the concentration of 10 ng/mL. * *p* < 0.05; ** *p* < 0.01; *** *p* ≤ 0.001.

We constructed a cell model of IUA using HESCs and used collagen I and fibronectin expression to verify the fibrosis phenotype. HESCs were stimulated with 0, 2.5, 5, and 10 ng/mL of TGF-β1 for 0, 12, 24, and 48 h; then, total protein was extracted, and a Western blot experiment was performed (Figure 3A,B). Compared with the normal control group, the expression of collagen I and fibronectin increased after TGF-β1 stimulation and showed concentration-dependent and temporal trends. When the cells were treated with 5 ng/mL and 10 ng/mL, the expression levels of collagen I and fibronectin were significantly increased, and the effect of inducing fibrosis was most obvious when the cells were treated with 10 ng/mL of TGF-β1 for 48 h. Therefore, in the following experiments, 10 ng/mL of TGF-β1 was used to stimulate HESCs for 48 h to induce fibrosis, and a cell model of IUA was constructed.

At the same time, we detected the expression of KLB protein using Western blotting (Figure 3C,D). The results showed that when HESCs were stimulated with TGF-β1 for 48 h, the expression of KLB protein increased as the concentration of TGF-β1 increased, and there was a statistical difference at 10 ng/mL. Meanwhile, the expression of KLB protein was not significantly increased 12 h and 24 h after stimulation with 10 ng/mL of TGF-β1. In conclusion, when HESCs were stimulated with TGF-β1 at a concentration of 10 ng/mL for 48 h, the expression of KLB protein was statistically significantly increased. 

### 2.4. KLB Promotes ESCs Proliferation and Fibrosis Induced by TGF-β1

**Figure 4:**
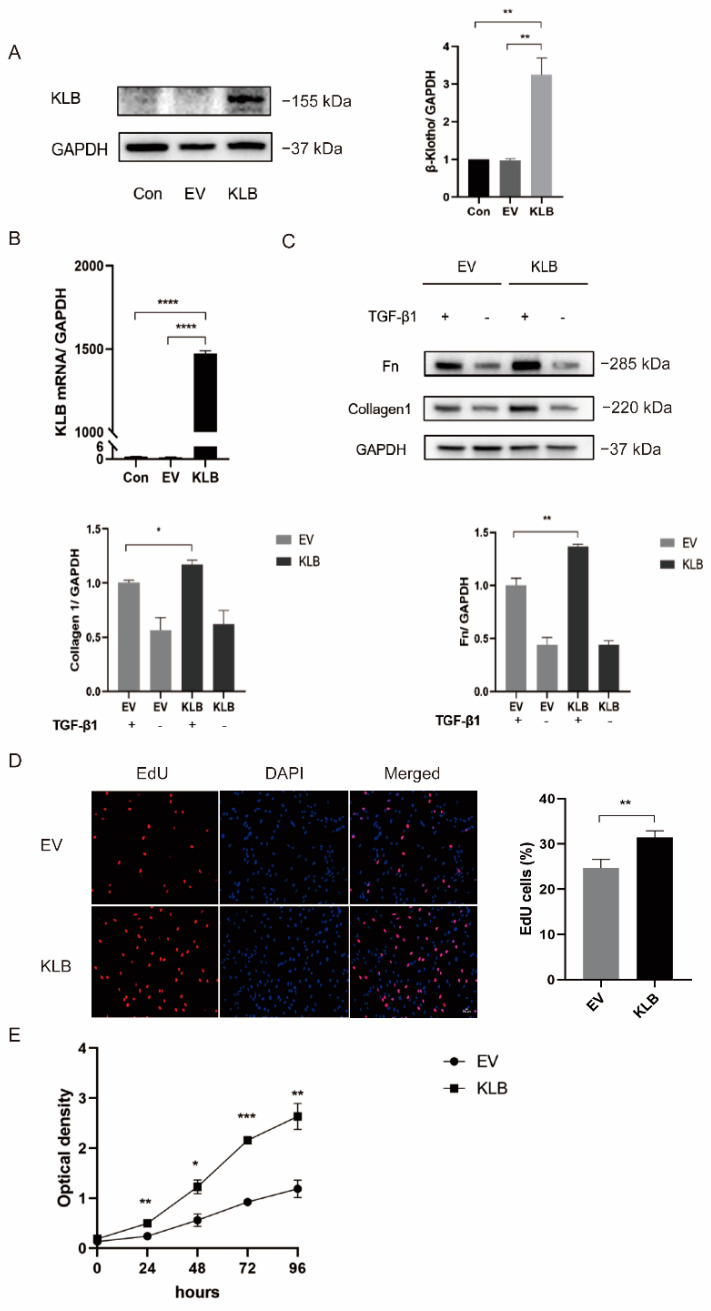
KLB can promote HESC fibrosis and cell proliferation. (**A**,**B**) Western blot and RT-qPCR analysis of KLB protein and mRNA levels in KLB-overexpressing ESCs. (**C**) Western blot showing that the expression of the fibrosis markers Collagen I and FN in the KLB overexpression group was significantly higher than that in the control group transfected with the empty plasmid under the same stimulation of TGF-β1. (**D**,**E**) EdU and CCK8 assays showed that the overexpression of KLB promoted the proliferation of HESCs. * *p* < 0.05; ** *p* < 0.01; *** *p* ≤ 0.001; **** *p* ≤ 0.0001.

We transfected HESCs with an empty plasmid and a KLB plasmid. After verifying the transfection efficiency of the plasmid by Western Blot (Figure 4A) and RT-qPCR (Figure 4B), we stimulated the experimental group with TGF-β1 at a concentration of 10 ng/mL. The results showed that the expression levels of Collagen I and Fibronectin were significantly increased in the TGF-β1-stimulated group compared with the group without TGF-β1 stimulation. Meanwhile, under the same stimulation with TGF-β1, the expression of the fibrosis markers Collagen I and Fibronectin in KLB-overexpressing cells was significantly higher than that in the control group (cells transfected with the empty plasmid), which proved that KLB could induce fibrosis in HESCs (Figure 4C).

The proliferation rate of HESCs was detected by a EdU experiment (Figure 4D). It was found that the proliferation rate of HESCs in KLB-overexpressing cells was 31.4%, while in the control group it was 24.6%, showing a statistically significant difference. The effect of KLB on the proliferation curve of HESCs was detected by the CCK8 assay (Figure 4E). It was found that the growth rate of HESCs-overexpressing KLB was significantly higher than that of the control group. Both experiments demonstrated that the overexpression of KLB could significantly increase the proliferation level of HESCs.

### 2.5. KLB Activates the PI3K/AKT Signaling Pathway in ESCs

**Figure 5:**
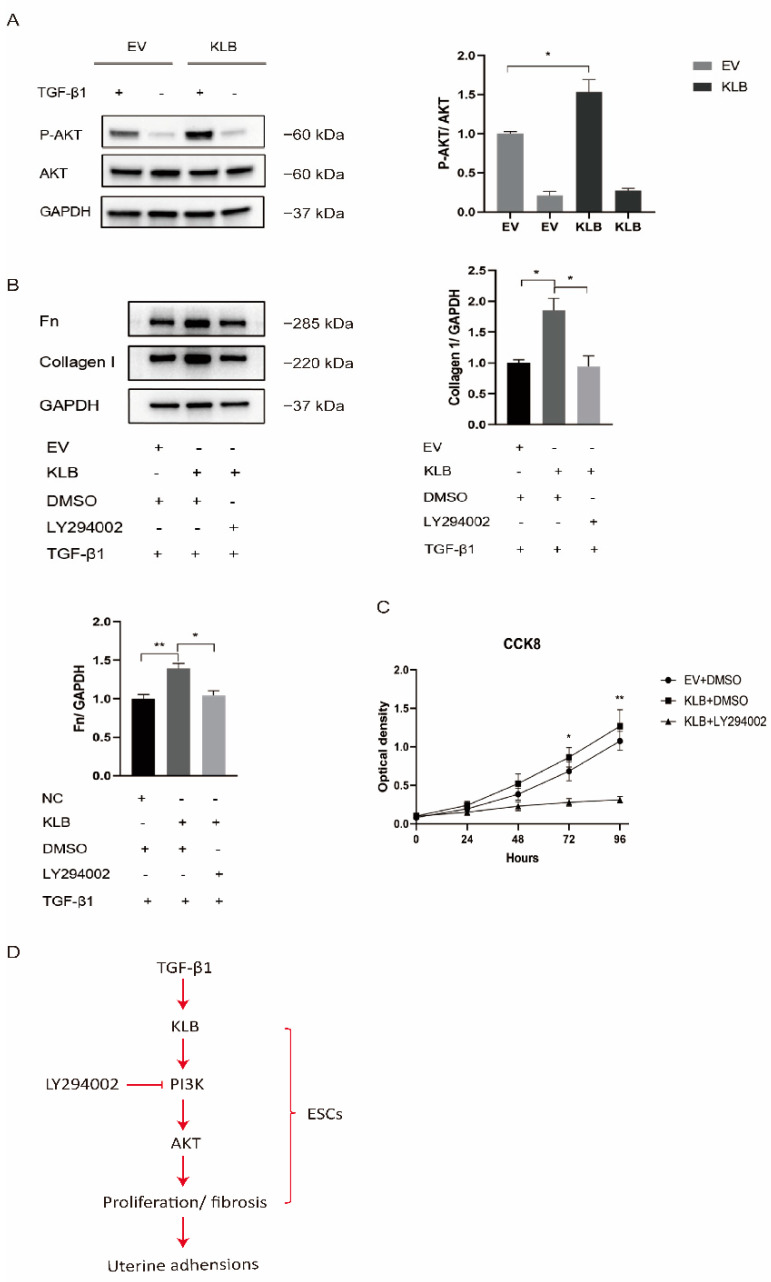
KLB promotes the fibrosis and proliferation of HESCs through the PI3K/AKT signaling pathway. (**A**) Through Western blot analysis, it was found that under the same stimulation with TGF-β1, the expression of P-AKT in the KLB overexpression group was significantly higher than in the control group transfected with the empty plasmid. (**B**) Through Western blot analysis, it was found that LY294002, an inhibitor of PI3K, could rescue the protein levels of Collagen I and FN after KLB overexpression. (**C**) The cell proliferation ability was detected by the CCK8 assay, and it was found that LY294002 could also reduce the cell proliferation promoted by KLB overexpression. (**D**) The activation the PI3K/AKT signaling pathway by KLB plays a critical role in IUA by regulating ESC proliferation and fibrosis. * *p* < 0.05; ** *p* < 0.01.

Under stimulation with TGF-β1, the activation of the PI3K/AKT signaling pathway in HESCs transfected with the empty plasmid and the KLB plasmid was compared. We found that TGF-β1 stimulation significantly activated the PI3K/AKT signaling pathway in HESCs, and under the same intensity of TGF-β1 stimulation, the expression of P-AKT in KLB-overexpressing cells was significantly higher than in the control group (Figure 5A). It was demonstrated that KLB overexpression could promote the activation of the PI3K/AKT signaling pathway stimulated by TGF-β1.

HESCs were treated with the PI3K/AKT pathway inhibitor LY294002 in a rescue experiment. As LY294002 was dissolved in DMSO, we used the same amount of DMSO as a control. After stimulating HESCs with TGF-β1 at a concentration of 10 ng/mL, we found that the expression of Collagen I and Fibronectin proteins in the KLB overexpression group was significantly higher than in the empty-plasmid group, while LY294002, an inhibitor of PI3K, could rescue the level of Collagen I and Fibronectin proteins after KLB overexpression (Figure 5B).

At the same time, the CCK8 experiment proved that LY294002 could also reduce the cell proliferation capacity increased by KLB overexpression (Figure 5C). The above experiments showed that KLB could enhance the fibrosis and proliferation ability of ESCs by activating the PI3K/AKT pathway, which could be reduced by LY294002, an inhibitor of the PI3K/AKT pathway (Figure 5D).

## 3. Discussion

Our studies have shown that KLB protein is highly expressed in the ESCs of patients with IUA and can promote cell proliferation and fibrosis by activating the PI3K/AKT pathway, thus inducing and mediating the development of IUA.

IUA and its severe forms, such as the Asherman syndrome, have complex etiologies and are often associated with severe clinical problems such as amenorrhea, infertility, and adverse reproductive outcomes [25]. Surgical intervention is often required for the treatment of IUA [26], but a recent multicenter randomized controlled study has shown that women diagnosed with IUA can still experience impaired reproductive outcomes even if they are treated [27]. Therefore, for women with or who may suffer from IUA, it is of great practical significance to deeply study the mechanisms of the occurrence and development of IUA in order to find the molecules and pathways that play a key role in endometrial fibrosis and to explore important therapeutic targets that can improve the symptoms of IUA.

The endometrium can be considered a unique model of wound healing because it can be repaired repeatedly without scarring or loss of function [28]. However, the maintenance of the endometrial biological function requires at least three key factors: (1) limiting the development of inflammation to prevent excessive tissue destruction; (2) the activation of stem cells for endometrial regeneration; (3) the scarless repair of the endometrium after menstruation [29]. We carried out related research on stromal cell fibrosis after endometrial injury and found that KLB plays an important role in endometrial fibrosis and can mediate the development of IUA, so we believe that KLB is a potential target for the treatment or prophylactic treatment of IUA.

KLB has a significant correlation with fibrosis. Thus far, there have been many studies on the pathogenesis of IUA, but the role of KLB in this disease has not been clear. Previous studies have shown that KLB is necessary for FGF19 and FGF21 to bind to their homologous receptors with high affinity and is associated with a variety of physiological activities and diseases [30,31]. Immunohistochemistry, immunofluorescence, and Western blot experiments demonstrated that KLB protein is significantly overexpressed in the ESCs of IUA patients. On the other hand, the expression of KLB and Fibronectin in the primary ESCs of IUA patients and normal women is significantly correlated, suggesting that cells with a higher fibrosis level can express KLB. However, these experiments did not clarify the role of KLB in the fibrosis process. To this end, we used the IUA cell model established by TGF-β1 to carry out further research.

TGF-β1 is a major fibrogenic cytokine that can regulate cell activation and ECM homeostasis in a variety of organs and tissues, thus playing a core role in the development of fibrosis. It can also participate in endometrial fibrosis and promote the formation of IUA [32,33]. Similar to the liver, kidney, and other organs, endometrial fibrosis is also a scar formation process. On the one hand, it is related to an increase in ECM deposition [34]. On the other hand, ESCs can be transformed into myofibroblasts under stimulation with TGF-β1 and participate in endometrial fibrosis [35]. A number of studies have reported that the presence of the fibrosis marker TGF-β1, which is highly expressed in the endometrial tissues of IUA patients or animal models, is closely related to the occurrence and development of IUA and is regarded as an early risk factor for disease recurrence [36]. The high expression of collagen I, Fibronectin, and KLB was evident in the IUA cell model established by TGF- β1, which proved that KLB was involved in the process of fibrosis.

At the same time, our study showed that the overexpression of KLB significantly increased HESC proliferation and promoted TGF-β1-induced cell fibrosis, suggesting that a high expression of KLB may participate in the process of endometrial fibrosis by promoting the proliferation and activation of HESCs.

TGF-β1-mediated cell fibrosis may be regulated by Smad-dependent and independent pathways. The TGF-β1/Smad pathway has long been considered a key driving factor in the pathogenesis of IUA, and Smad3 has been proven to be involved in Collagen gene transcription and protein expression induced by TGF-β1 [37,38]. It has been reported that the secreted Klotho protein inhibits TGF-β1/Smad signaling and thus suppresses renal fibrosis and tumor metastasis in mice [39]. However, the participation and roles of the Smad-independent pathways in ESC fibrosis mediated by TGF-β1 need to be deeply studied. The PI3K/AKT pathway has been found to play an important role in lung, liver, pancreas, and other organs’ fibrosis [40,41,42]. Our study found that the PI3K/AKT pathway can be activated by TGF-β1 and participate in endometrial fibrosis. Meanwhile, our study also confirmed that KLB overexpression can promote the activation of the PI3K/AKT signaling pathway stimulated by TGF-β1 and thus enhance cell fibrosis and proliferation. In order to clarify the role of the PI3K/AKT pathway in KLB-mediated induction of cell fibrosis, we carried out rescue experiments. The results showed that LY294002, an inhibitor of the PI3K/AKT pathway, could inhibit the cell fibrosis and cell proliferation mediated by KLB overexpression, which proved that KLB could indeed act through the PI3K/AKT pathway. The ectopic lesions in endometriosis patients constantly bleed, constantly repair, and gradually form fibrosis. Therefore, endometriosis is a progressive fibrotic process in which molecular biology changes play a major role. It should be noted that this study has potential additional implications regarding fibrosis associated with endometriosis. There are also some limitations in our research. The endometrial tissue we obtained was exfoliated by patients during hysteroscopic surgery. For IUA patients, the endometrium is very precious and rare, so the sample size was small, including endometrial tissue from 10 IUA patients and 10 normal people. However, we established an animal model of IUA, and KLB was also found to be highly expressed in the stromal cells of IUA rats. Further in vivo studies are needed in the future.

## 4. Materials and Methods

### 4.1. Human Endometrial Tissue Collection

This study was approved by the Hospital Ethics Committee (No. 2018-47). Due to the involvement of human specimens, each patient signed an informed consent form before we obtained the specimen. The endometrial tissue of the study group came from IUA patients who underwent hysteroscopic surgery in the Reproductive Hospital-affiliated Shandong University. In the control group, the endometrial tissue was exfoliated during operation in infertile patients without uterine cavity disease who were examined by hysteroscopy before in vitro fertilization/intracytoplasmic sperm injection (IVF/ICSI) treatment. None of the patients and controls we included had submucous uterine fibroids, uterine malformations, endometrial polyps, or endometriosis. The endometrial samples were obtained in the follicular phase of the menstrual cycle.

### 4.2. Animal Model

In this study, a rat model of IUA was established by mechanical injury. Ten–twelve-week-old female SD rats were anesthetized by the intraperitoneal injection of 1.5% pentobarbital sodium solution, and a median incision was made along the midline of the abdominal wall, with a length of about 2.5 cm. We cut the skin, muscles, and peritoneum to expose the uterus. After exposing the right uterine horn, a 1.5 cm longitudinal incision was made along the long axis of one uterine horn toward the ovary, starting at 0.5 cm from the uterine bifurcation. The uterine cavity was exposed, and the endometrial layer was scraped with a surgical blade. The contralateral left uterus was used as a control. After curettage, we rinsed the uterine cavity and abdominal cavity with aseptic saline and absorbed the excess fluid with sterile gauze. After suturing according to the anatomical structure, the rats were sent back to the cage. Eight days after the operation, the uteruses were collected, and the endometrium was evaluated by immunohistochemistry.

### 4.3. Immunofluorescence and Immunohistochemistry

The immunofluorescence and immunohistochemistry protocols were similar to those detailed in the articles we published before [43]. For immunofluorescence, paraffin sections of the endometrium were dewaxed and hydrated, before antigen retrieval was performed with Ethylene Diamine Tetraacetic Acid (EDTA). The sections were incubated with an endogenous peroxidase blocking solution, blocked with goat serum, and then incubated with a primary antibody against KLB (1:200, bs-16748R, Bioss, Beijing, China) at 4 °C overnight. The next day, the sections were incubated with secondary fluorescent antibodies (1:500, A-11012, Invitrogen, Waltham, MA, USA) for 1 h in the dark and at room temperature. We added an anti-quenching tablet containing 4′,6′-diamidino-2-phenylindole (DAPI) to the paraffin sections and incubated them at room temperature for 1 h. For the immunohistochemistry of endometrial tissue in rats, the paraffin sections were blocked with goat serum for 1 h and incubated with the primary antibody against KLB (1:50, A15629, ABclonal, Wuhan, China). The secondary antibody, labeled with horseradish peroxidase (HRP), was added, followed by a freshly prepared diaminobenzidine (DAB) chromogenic solution. Finally, the sections were photographed with an Olympus microscope.

### 4.4. Quantitative Real-Time PCR (qRT-PCR) Analysis

Total RNA was extracted from the tissues or cells using the TRIzol reagent (Invitrogen, USA). The method was reported in detail in previously published articles [44]. Total RNA (1 μg) was reversely transcribed using the Prime Script RT Reagent Kit with gDNA Eraser (TaKaRa, Tokyo, Japan). The SYBR Premix kit (TaKaRa, Japan) was used for RT-qPCR. Glyceraldehyde-3-Phosphate Dehydrogenase (GAPDH) was used as the internal control. The results were analyzed with the 2-ΔΔCt method; the sequences of the PCR primers are listed in Table 1.

### 4.5. Western Blot Analysis

The RIPA lysis buffer containing protease/phosphatase inhibitor (5872, Cell Signaling Technology, Danvers, MA, USA) was used to extract total protein from tissue or cells. The Pierce BCA Protein Assay Kit (23225, Thermo Scientific, Waltham, MA, USA) was used for the protein quantitative analysis. We used a 4–20% concentration-gradient Smart PAGE Precast Protein Gel (SLE009, Smart-Lifesciences, Changzhou, China) for protein separation; the proteins were transferred to 0.45 µm polyvinylidene fluoride (PVDF) membranes. The membranes were blocked in 5% skim milk at room temperature for 1 h and incubated at 4 °C overnight with primary antibodies against KLB (1:1000, ab106794, Abcam, UK), Collagen I (1:1000, ab260043, Abcam, UK), Fibronectin (1:1000, ab268020, Abcam, UK), Phospho-AKT (1:1000, CST, 9271, USA), AKT (1:1000, CST, 9272, USA), or GAPDH (1:10,000, 60004-1-Ig, Proteintech, Chicago, IL, USA). After each sample was washed with 1X Tris-buffered saline–Tween (TBST) 3 times, each sample was incubated at room temperature for 1 h with goat anti-rabbit and goat anti-mouse HRP-conjugated secondary antibodies. We washed each sample 3 times again with TBST. Finally, the enhanced chemiluminescence (ECL) detection system was used to detect the proteins. GAPDH was used as a reference control, and the Image Lab software was used for the semi-quantitative analysis of each protein band.

### 4.6. Primary Human Endometrial Stromal Cell (ESC) Isolation and Culture

Primary ESCs were isolated from fresh endometrial tissue, and the tissue was washed repeatedly with PBS buffer to remove blood clots as much as possible. We used ophthalmic scissors to cut up the endometrial tissue roughly into a paste. The pasted tissue was poured into a 15 mL centrifuge tube, and 4 mL of 0.25% collagenase was added. After full shock and mixing, the mixture was transferred into a 37 °C water bath pot for 40 min and shaken every 6–7 min. After 40 min, we extracted the centrifuge tube from the water bath pot and added 3 times the tissue volume of PBS buffer to stop the protein digestion. The digested tissue was sequentially passed through metal screens of 100 mesh and 400 mesh and cleaned with PBS buffer to remove impurities and collect the filtered components. After centrifugation at 1000 rpm/min for 5 min, the supernatant was removed, and the cells in the lower layer were isolated as ESCs.

The immortalized human endometrial stromal cell line (HESC) was presented by Prof. Junhao Yan (Shandong University, Jinan, China).

### 4.7. Cell Culture

HESCs and primary ESCs were cultured in DMEM/F-12 without phenol red (Gibco, Waltham, MA, USA), supplemented with 10% charcoal-stripped FBS (Biological Industries, Israel). The cells were cultured at 37 °C in a 5% CO_2_ atmosphere. To avoid serum interference, the cells were starved in DMEM/F12 containing 0.1% FBS for 12 h before adding TGF-β1 (100-21C, Peprotech, Rocky Hill, NJ, USA) to induce cell fibrosis. The PI3K inhibitor LY294002 (15 μM, MCE, USA) was pretreated for 1 h before use.

### 4.8. Plasmid Transfection

The KLB plasmid and a control empty plasmid were purchased from GENECHEM Co., Ltd. (Shanghai, China). When 70–80% confluence of HESCs was reached, transfection was performed with Lipofectamine 3000 (Invitrogen, USA) according to the manufacturer’s instructions. The transfection efficiency was evaluated by RT-qPCR or Western blot.

### 4.9. Cell Counting Kit-8 (CCK8) Assay

HESCs were transfected with the specified plasmid. After 24 h, HESCs were inoculated in 96-well plates with 2 × 103 cells in 100 μL medium per well, and three repeated wells were set. After 0, 24, 48, 72, and 96 h of incubation, 10 μL of CCK8 (Beyotime, Haimen, China) was added to each hole, and incubation was performed at 37 °C for 2 h. Absorbance (optical density) at 450 nm was measured by a microplate reader, and a growth curve was drawn.

### 4.10. Assay Based on 5-Ethyl-2′-deoxyuridine (EdU) 

According to the instructions of the Cell-Light EdU Apollo567 In Vitro Imaging Kit (RiboBio, Guangzhou, China), we incubated HESCs and an EdU solution at 37 °C for 2 h, then fixed the cells in 4% paraformaldehyde at room temperature for 30 min. After adding the Apollo reaction reagent and incubating it with HESCs for 30 min, DNA was stained with Hoechst33342. Images were taken using the Olympus microscope; the nucleus showed blue fluorescence, and the cells labeled with EdU showed red fluorescence. A cell count was carried out, and the red/blue ratio indicated the rate of cell proliferation.

### 4.11. Statistical Analysis

Continuous variables were expressed as mean ± SEM. If each group satisfied a normal distribution and the inter-group variance was homogeneous, an independent sample *t*-test was used for the comparison between the two groups, and a one-way ANOVA was used for multi-group comparisons. If the distribution was not normal, the nonparametric Mann–Whitney U test was used for the comparison between the two independent groups, and the Kruskal–Wallis H test was used for the comparison between multiple groups.

The GraphPad Prism 8.0 and SPSS 24.0 software packages were used to make statistical charts and conduct statistical analyses. All experiments were independently repeated three times, and the difference was considered statistically significant when *p* < 0.05.

## 5. Conclusions

To sum up, our study found that KLB can participate in endometrial fibrosis and promote IUA by activating the PI3K/AKT pathway. A treatment that reduces the activation of KLB and the PI3K/AKT pathway may inhibit the transformation of endometrial stromal fibroblasts into myofibroblasts, thereby reducing fibrosis and scar formation. This study may provide a direction for the treatment of IUA.

## Figures and Tables

**Table 1:** Primer sequences.

Gene	Forward Sequence	Reverse Sequence
Homo-KLB	GCAGTCAGACCCAAGAAAA TAC	GAGTAGAACCAGGGTGGAGAA
Homo-Collagen I	TAAAGGGTCACCGTGGCTTC	GGGAGACCGTTGAGTCCA TC

## Data Availability

All the data in this study are included in this manuscript.
